# Trouble in the Camp

**Published:** 1890-05

**Authors:** B. H. Catching

**Affiliations:** Atlanta, Georgia


### CORRESPONDENCE.

### Trouble in the Camp.

ATLANTA, GEORGIA.

*Dear Doctor Taft:*—

It seems all is not serene with dental college matters in this city. One of the professors is now an ex-professor with his feelings very much hurt and his pocketbook wounded altogether to the tune of five thousand dollars—so the court docket says. Ex-Professor Henley claims, in his bill filed against said college, that he was one of the prime movers in starting the institution, putting both time and money in it, expecting to get the latter back with big interest, in the future, but before such expectations were realized he was made an ex-professor with no stipend or annuity. He does not set forth why he was shelved but leaves us to guess. So I guess the division of the spoils was too long—the shorter method being preferable. Any way the ex-professor is asking the laws of the Commonwealth to hear his grievances and right his wrongs. Will it do it? If it goes before an ordinary jury, and the school is proven to be a close corporation, it will suffer and the ex-professor will be happy.

I see Germany is playing havoc with the American degree. They must compare it to the American hog, which was knocked out of that empire some years ago. Chicago must go the whole hog or none. She has fifteen dental colleges, each with a *dean* and a corps of professors. She packs more hogs and issues more diplomas than any city in America. Germany must have a spite against Chicago, and Herod-like, sweep the whole country to get the right ones. You know, doctor, all our schools are first-class, all belong to the College Association, all issue diplomas on merit, caring nothing for the number of matriculates or the money; just doing it for glory. The Georgia Dental Board convened here two weeks ago and remanded back to school seventeen per cent. of the applicants, one from the home school, and one from one of the Baltimore schools. We have a good, fearless board of examiners. The questions this year were not difficult. A student just from college who could not answer them, ninety per cent. at least, deserved to be thrown.

Some of us have made up our minds about the Independent Congress, New Jersey has called to meet in Chicago in '92. If Chicago wanted an invitation issued why in the world didn't it get some State to do it that has more squaie miles than New Jersey? Brother Stockton, good fellow that he is, will have to call louder if he wants to be heard over this broad land. We don't need a congress and we won't have one. We can, and should have during the fair, if there be a fair, a big reunion meeting and invite as many to the feast from across the pond as will come, but not invite them to a congress; simply to an American dental love feast.

The DENTAL REGISTER presented us one of the greatest anomalies in its last issue. It was in the shape of W. Storer How reading the dental profession a lecture on ethics. It is intended to be read between the lines. You can't bring out another anomaly so great.

B. H. CATCHING.

